# Frontopolar cortex and decision-making efficiency: comparing brain activity of experts with different professional background during an exploration-exploitation task

**DOI:** 10.3389/fnhum.2013.00927

**Published:** 2014-01-22

**Authors:** Daniella Laureiro-Martínez, Nicola Canessa, Stefano Brusoni, Maurizio Zollo, Todd Hare, Federica Alemanno, Stefano F. Cappa

**Affiliations:** ^1^Department of Management, Technology, and EconomicsETH Zurich, Zurich, Switzerland; ^2^Department of Management and Technology, Bocconi UniversityMilan, Italy; ^3^Center for Cognitive Neuroscience & CERMAC, Vita-Salute San Raffaele UniversityMilan, Italy; ^4^Division of Neuroscience, San Raffaele Scientific InstituteMilan, Italy; ^5^KITeS, Department of Management and Technology, Bocconi UniversityMilan, Italy; ^6^Center for Research in Innovation, Organization and Strategy (CRIOS), Department of Management and Technology, Bocconi UniversityMilan, Italy; ^7^Laboratory for Social and Neural Systems Research, Department of Economics, University of ZurichZurich, Switzerland

**Keywords:** decision-making, efficiency, exploration-exploitation, *f*MRI, frontopolar cortex

## Abstract

An optimal balance between efficient exploitation of available resources and creative exploration of alternatives is critical for adaptation and survival. Previous studies associated these behavioral drives with, respectively, the dopaminergic mesocorticolimbic system and frontopolar-intraparietal networks. We study the activation of these systems in two age and gender-matched groups of experienced decision-makers differing in prior professional background, with the aim to understand the neural bases of individual differences in decision-making efficiency (performance divided by response time). We compare brain activity of entrepreneurs (who currently manage the organization they founded based on their venture idea) and managers (who are constantly involved in making strategic decisions but have no venture experience) engaged in a gambling-task assessing exploitative vs. explorative decision-making. Compared with managers, entrepreneurs showed higher decision-making efficiency, and a stronger activation in regions of frontopolar cortex (FPC) previously associated with explorative choice. Moreover, activity across a network of regions previously linked to explore/exploit tradeoffs explained individual differences in choice efficiency. These results suggest new avenues for the study of individual differences in the neural antecedents of efficient decision-making.

## Introduction

Adaptive behavior in an uncertain world requires managing the trade-off between exploiting known sources of reward and exploring the environment to gather information about different, potentially more valuable options. The ability to achieve an optimal or approximately optimal strategy between exploration and exploitation has been extensively investigated in the context of foraging studies in animals (Stephens and Krebs, 1986). In particular, model-based approaches allow a computational analysis of exploration strategies underlying the individuals' behavioral choices in human gambling tasks (Daw et al., 2006). At the neural level, exploitative choices engage the dopaminergic frontolimbic-striatal system projecting to the ventromedial prefrontal cortex (Beeler et al., 2010), associated with reward experience and anticipation (Tobler et al., 2007). Explorative choices, instead, engage the intraparietal sulcus (IPS) and lateral prefrontal regions, particularly frontopolar cortex (FPC) (Daw et al., 2006), with activity in the latter region predicting effective switching between exploitation and exploration (Boorman et al., 2009). In support to this distinction, polymorphisms in genes controlling striatal and prefrontal dopaminergic functions have been associated with, respectively, individual differences in exploitation and exploration (Frank et al., 2009). Also noradrenergic projections from the locus coeruleus have been associated with the modulation of explorative behavior, although from variable perspectives (e.g., Aston-Jones and Cohen, 2005; Yu and Dayan, 2005; Cohen et al., 2007; Good and Michel, 2013; see Discussion).

These studies provide clues about the role of individual differences in choice efficiency in complex, competitive, and fast-changing decision settings. Individual differences in decision-making are important not only for the individuals themselves, but also the groups and organizations in which they function. Indeed, research in management sciences has shown that adequately managing the balance between searching for radical innovations (exploration) and maintaining or improving existing processes (exploitation) is crucial for an organization's adaptation and survival (March, 1991; Good et al., 2001; Benner and Tushman, 2003). In addition to the quality of a decision, speed is also a crucial strategic variable. Organizations differ in the speed with which they explore different options, generate alternatives, and exploit specific strategies (Eisenhardt and Bourgeois, 1988; Eisenhardt, 1989). The ability to make decisions quickly in order to keep up with fast environmental changes is fundamental to survival and market performance. First mover advantages (i.e., the leadership position gained by the initial occupant of a market) have been shown to provide long-term benefits to those organizations that enter and exploit new markets earlier than potential competitors (Gort and Klepper, 1982; Glazer, 1985). In some circumstances, speed might even be more beneficial than technical performance *per se* (Eisenhardt, 1989; Brusoni et al., 2007; Hawk et al., 2013). However, it is clearly optimal to maintain both quality and speed in the decision-making process (Yu et al., 2011; Tzovara et al., 2012; Symmonds et al., 2013). Individuals, and the organizations they lead, will perform best if they can establish an efficient decision process that minimizes the well-documented inverse relation between speed and performance (i.e., speed-accuracy tradeoff) (Fitts, 1954; Wickelgren, 1977; Luce, 1991; Bogacz et al., 2010), thereby generating fast responses that still maintain adequate performance.

Here we examine neurobiological mechanisms that promote fast and accurate (i.e., efficient) decisions when faced with exploitative vs. explorative choice options. We study decision-making efficiency, operationalized as total payoff divided by response time, in two age and gender-matched groups of experienced decision-makers. Our hypothesis is that, while engaged in a task requiring fast and efficient decision-making, individuals with experience in facing a broad range of pressing, heterogeneous, decisions, compared with a group experienced in making more specialized choices, will show better performance. Moreover, we predict that this efficient performance will be driven by regions previously associated with exploratory choices and effective behavioral switching, such as FPC (Daw et al., 2006; Boorman et al., 2009), anterior cingulate cortex (Kolling et al., 2012) and locus coeruleus (Aston-Jones and Cohen, 2005).

Entrepreneurs are an ideal population in which to examine decision efficiency, as during the start-up phase they continuously face many heterogeneous decisions that need immediate attention (Shane and Venkataraman, 2000; Shane, 2006; Ács and Audretsch, 2010). Since entrepreneurs cannot rely on an established organizational structure that allows them to specialize, they need to combine creativity to explore and generate ideas with the effort necessary to turn them into marketable products and exploit their outcomes. The early period after start-up is characterized by a very high organizational mortality rate. Research in sociology and economics has found that entrepreneurs' characteristics (e.g., education, industry experience) are an important predictor of start-up's survival (Evans and Leighton, 1989; Brüderl et al., 1992; Klepper and Simons, 2000; Fairlie, 2002; Klepper, 2002). However, these studies have little to say about the cognitive processes that successful entrepreneurs deploy to make decisions, although one preliminary behavioral study focusing on risk-taking showed increased risk-propensity and higher scores for impulsiveness and cognitive flexibility in entrepreneurs compared to a control group of business managers (Lawrence et al., 2008). Accordingly, we capitalized on the distinctive features of entrepreneurs to investigate the neural and cognitive bases of the ability to efficiently make decisions in an exploration-exploitation game. Managers in established companies are an ideal comparison group for our purposes, because they typically can specialize (by skill, unit, or function) and focus on efficiency improvements within their specialty.

Given the established track record of successful decision-making in both groups, we expected that all subjects would utilize effective choice strategies to maximize profits in this simple task. However, we also theorized that the characteristics and experience of successful entrepreneurs would allow result in greater efficiency for this group compared to the managerial group. At the neural level, we expected differences in efficiency to be related to brain activity in regions previously associated with exploratory choices and effective behavioral switching, such as FPC (Daw et al., 2006; Boorman et al., 2009), anterior cingulate cortex (Kolling et al., 2012) and locus coeruleus (Aston-Jones and Cohen, 2005).

## Materials and methods

### Subjects and experimental design

In order to test our hypothesis we studied two age and gender-matched groups of individuals with similar personal background (i.e., place of origin and schooling level), but different professional experience, such as managers and entrepreneurs. We collected behavioral and functional-Magnetic-Resonance-Imaging (*f*MRI) data from 50 right-handed (Oldfield, 1971) healthy subjects [11 females; females' mean age = 33.182 years, standard deviation (*SD*) = 6.290; males' mean age = 35.589 years, *SD* = 6.965]. Based on strict criteria about their expertise and the history of their achievements, they were assigned to 2 groups: 24 “entrepreneurs” (mean age = 35.5 years, *SD* = 6.467; 4 females) and 26 “managers” (mean age = 34.654 years, *SD* = 7.261; 7 females).

Inclusion criteria for all the subjects in the group “entrepreneurs” were the requirement to have founded an organization that was initially established based on their venture idea. Besides, they all had implemented their idea and were—at the time of the study—running the organization, being constantly involved in various pressing heterogeneous strategic decisions related to administrative areas as diverse as marketing, human resources, production, research and development, and finance. Inclusion criteria for being classified as a “manager” required the individual to be working inside an organization and being constantly involved in strategic decisions in their areas of expertise (e.g., automotive technologies), and had no venture experience. For a summary of these criteria see Table 1. It is important to note that the two groups did not differ in measures of general intelligence (Raven's progressive matrices score for managers = 6.38, entrepreneurs = 6.04, *p*−value = 0.6843). All subjects had normal or corrected-to-normal visual acuity. None reported a history of psychiatric or neurological disorders, nor current use of psychoactive medications. They gave their written informed consent to the experimental procedure, which had been approved by the local Ethics Committee.

**Table 1:** **Participants' selection criteria**.

**Group managers**	**Group entrepreneurs**
Matched by age, gender, place of origin and schooling level	
Responsible for leading a group of at least 2 individuals	
Makes decisions related to: human resources *or* marketing *or* budget allocation and finance	Makes decisions related to: human resources, *and* marketing, *and* budget allocation and finance
–Does not apply–	Has worked in the organization s/he created for at least 3 years (founded it and the organization exists for at least 3 years)

*The first two rows show the criteria shared by all participants. The last two rows show the criteria that differentiated the entrepreneurs from the managers*.

### Task and procedure

**Figure 1:**
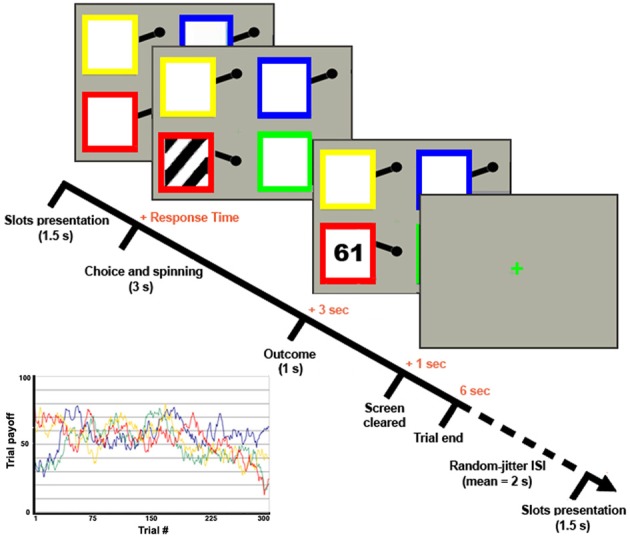
**4-armed bandit task**. Graphical representation of the 4-armed bandit task and of the slots' payoff functions [following the task design of Daw et al. (2006)].

The two groups played a 4-armed bandit task, a classical task of exploitative/explorative decision-making (Daw et al., 2006) (Figure 1). The task involved repeated choices among four differently colored slot machines that lead to variable gains in successive trials all having the same structure and lasting 6 s. The slots were shown for 1.5 s, during which subjects had to indicate the chosen one by pressing with the right hand the corresponding button of a response box. Responses given within this period started the animation of the slot machine (e.g., rotation of the spinning wheels for 3 s), after which the payoff was shown for 1 s. If no choice was given within the slots-presentation period, a red X was displayed until the end of the trial. Trials were separated by a green “+,” whose duration was varied (“jittered”) in every trial in order to desynchronize the timings of event-types with respect to the acquisition of single slices within functional volumes and to optimize statistical efficiency (Dale, 1999). The OptSeq2 Toolbox (http://surfer.nmr.mgh.harvard.edu/optseq/) was used to estimate the optimal ISIs (mean *ISI* = 2 s, *SD* = 1.987 s, range = 0–10 s). Subjects played 300 trials overall, subdivided in 4 *f*MRI-runs of 75 trials each. The payoff structure was the same as in Daw et al. (2006). Namely, the payoff for choosing the *i*th slot machine on trial *t* was between 1 and 100 points, drawn from a Gaussian distribution (standard deviation σ_*o*_ = 4) around a mean μ_*i,t*_ and rounded to the nearest integer. At each time-step, the means diffused in a decaying Gaussian random walk, with μ_*i,t* + 1_ = λ_μ *i,t*_ + (1 − λ)θ + ν for each *i*. The decay parameter λ was 0.9836, the decay center θ was 50, and the diffusion noise ν was zero-mean Gaussian with standard deviation σ_*d*_ = 2.8 (see Figure 1 for a graphical representation of the payoffs of the 4 slots in all the 300 trials).

Visual stimuli were viewed via a back-projection screen located in front of the scanner and a mirror placed on the head-coil. The software Presentation 14.4 (Neurobehavioral systems, Albany, CA, http://www.neurobs.com) was used both for stimulus presentation and subjects' answers recording.

All subjects underwent a training session preceding the functional acquisition, and were informed that they would be paid based on their overall earning in the whole study.

### Behavioral-data analysis

Following previous reports (Daw et al., 2006), we modeled participants' behavior in the 4-armed bandit task using a Kalman filtering algorithm and softmax choice rule to estimate participants' beliefs about the current value of each slot machine using maximum likelihood. The Kalman filter is a generalized temporal difference algorithm that tracks the uncertainty of rewards in addition to point estimates of their value at each point in time as a function of the past history of rewards. The best fitting model parameters were: σ_*o*_ = 0, λ = 0.8941, θ = 54.77, σ_*d*_ = 6.32, μ_*i*,0_^pre^ = 67.78, and σ_*i*,0_^2pre^ = 8.18, with the same initial mean and standard deviation assumed for all bandits *I* = 1:4. Note that these variables correspond to the reward generating process in the 4-armed bandit task described above. On the basis of this model, we assigned an estimated value to each slot machine for every trial. Exploitative choices were defined as those in which the slot machine currently believed to have the highest estimated value among all four options was selected. Explorative choices were defined as choices in which a slot machine other than the one believed to have the highest value at that point in time was selected. A summary of the results on the behavioral measures for all participants, managers and entrepreneurs is presented in Table 2.

**Table 2:** **Different measures of behavioral performance (efficiency, payoff, and RT)**.

**Variables**	***N***	**Mean**	***SD***	**Min**	**Max**
Efficiency	50	44291.6	9020.5	24517.73	76520.73
Payoff	50	18049.18	600.7963	15356	18795
Response time	50	0.422822	0.080769	0.239935	0.65741
Efficiency—all	50	44292	9021	24518	76521
Efficiency—managers	26	41625	10616	24518	76521
Efficiency—entrepreneurs	24	47180	5841	35844	58866
Payoff—all	50	18049	601	15356	18795
Payoff—managers	26	18003	682	15356	18774
Payoff—entrepreneurs	24	18099	508	16869	18795
Response time—all	50	0.423	0.081	0.240	0.657
Response time—managers	26	0.454	0.095	0.240	0.657
Response time— entrepreneurs	24	0.389	0.043	0.315	0.485

### *f*MRI-data acquisition

Anatomical T1-weighted and functional T2^*^-weighted MR images were acquired with a 3 Tesla Philips Achieva scanner (Philips Medical Systems, Best, NL), using an 8-channels Sense head coil (sense reduction factor = 2). Functional images (307 per run) were acquired using a T2^*^-weighted gradient-echo, echo-planar (EPI) pulse sequence [37 ascending transverse slices, *TR* = 2000 ms, *TE* = 30 ms, flip-angle = 85°, Field-Of-View (*FOV*) = 192 × 192 mm, slice-thickness = 3.4 mm, inter-slice gap = 0.2, in-plane resolution = 3 × 3 mm]. Due to specific hypotheses concerning the involvement of the ventromedial prefrontal cortex (vmPFC) in exploitative choice (Daw et al., 2006; Boorman et al., 2009), we tilted the FOV 30° downwards with respect to the bi-commissural line to reduce susceptibility artifacts from this region. While resulting in the loss of signal from the occipital cuneus in some subjects, this procedure significantly enhanced data-acquisition from one of our primary regions of interest close to air/tissue interfaces. Immediately after the functional scanning, a high-resolution T1-weighted anatomical scan (150 slices, *TR* = 600 ms, *TE* = 20 ms, slice-thickness = 1 mm, in-plane resolution = 1 × 1 mm) was acquired for each subject.

### *f*MRI-data pre-processing and statistical analysis

Image pre-processing and statistical analyses were performed using SPM8 (Wellcome Department of Cognitive Neurology, http://www.fil.ion.ucl.ac.uk/spm), implemented in Matlab v7.4 (Mathworks, Inc., Sherborn, MA) (Worsley and Friston, 1995). The first 6 volumes of each functional run were discarded to allow for T1 equilibration effects. All remaining 1228 volumes from each subject were then spatially realigned (Friston et al., 1996) to the first volume of the first run and unwarped (Andersson et al., 2001), spatially normalized and re-sampled in 2 × 2 × 2-mm voxels after normalization, and spatially smoothed with an 8-mm full-width half-maximum (FWHM) isotropic Gaussian kernel. The resulting time series across each voxel were then high-pass filtered to 1/128 Hz, and serial autocorrelations were modeled as an AR (1) process.

In the statistical analysis we focused on the regions showing significant changes in cerebral activity related to exploitative vs. explorative decision-making. Statistical parametric maps were generated using a random-effects model, implemented in a 2-level procedure (Friston et al., 1999).

The first (single-subject) level GLM included three regressors of interest: (1) Explore choices (as defined by computational analyses, see Behavioral-data analysis), (2) Exploit choices, and (3) Outcome (regardless of choice). An additional regressor modeled all other visual stimuli (i.e., the spinning of the wheel and those trials in which an out-of-time response or no response was given). Following Daw et al. (2006), the onset times for both Explore and Exploit choices were modeled as zero duration events time-locked to midway between the presentation of the bandits and the recorded key-press indicating choice of a specific bandit. All regressors were convolved with a canonical hemodynamic response function (HRF), and parameter estimates for all regressors were obtained by maximum-likelihood estimation.

At the second (group) level, we ran an analysis of variance (ANOVA) (Friston et al., 1999), employing a general statistical threshold of *p* < 0.05 corrected for multiple comparisons across all voxels in the brain using a False-Discovery-Rate procedure (Genovese et al., 2002). We assessed the main effects and interactions between the fixed factor “choice type” (exploitative-explorative) and the random factor “group” (managers-entrepreneurs) in a 2 × 2 full factorial model.

The location of the activation foci are reported in Table 3 in the stereotactic space of Talairach and Tournoux (1988) after correcting for differences between the latter and the MNI coordinate systems by means of a non-linear transformation (Brett et al., 2001). Those cerebral regions for which maps are provided were also localized with reference to cytoarchitectonical probabilistic maps of the human brain, using the SPM-Anatomy toolbox v1.8 (Eickhoff et al., 2005).

**Table 3:** **Neural correlates of exploitative vs. explorative choice**.

**K**	**H**	**Anatomical region**	**MNI**			**Voxel T-score**	**Cluster *p*-value**
			***X***	***Y***	***Z***		
**EXPLOIT > EXPLORE**							
5627	L	Superior frontal gyrus	−12	42	48	8.26	0.000
	L	Mid orbital gyrus	−4	56	−4	7.98	
	R	Anterior cingulate cortex	4	36	6	6.76	
	L	Anterior cingulate cortex	−6	32	−10	5.97	
3844	L	Middle temporal gyrus	−54	−6	−18	8.07	0.000
	L	Posterior cingulate cortex	−6	−46	30	7.44	
3473	R	Rolandic operculum OP4	60	−6	10	5.91	0.000
	R	Middle temporal gyrus	50	2	−28	5.83	
828	L	IFG (p. Triangularis) 45	−54	24	14	7.03	0.000
	L	IFG (p. Orbitalis)	−42	34	−12	6.72	
719	L	Paracentral lobule 6	−6	−24	62	4.97	0.000
	R	Postcentral gyrus 3b	16	−38	62	4.60	
599	L	Hippocampus (CA/SUB)	−24	−16	−14	7.06	0.000
487	R	Hippocampus (CA/SUB)	26	−16	−16	8.07	0.000
308	R	IFG (p. Orbitalis)	40	32	−16	6.52	0.000
136	R	Anterior insula/vmPFC	22	30	10	5.31	0.001
**EXPLORE > EXPLOIT**							
8631	R	Precuneus	10	−64	54	15.27	0.000
	L	Precuneus	−12	−66	60	13.37	
	L	Superior parietal lobule	−16	−70	54	12.51	
	R	Inferior parietal lobule	36	−42	46	11.3	
	L	Inferior parietal lobule	−36	−42	40	11.09	
	L	Superior parietal lobule 7PC	30	−48	46	10.46	
	R	Right supramarginal gyrus	40	−34	42	9.47	
4846	R	Superior frontal gyrus	24	0	56	12.98	0.000
	L	Superior frontal gyrus	−24	−4	56	11.33	
	L	SMA	−2	12	48	11.05	
	R	Middle cingulate cortex	8	22	34	7.93	
	L	FPC-middle frontal gyrus	−36	50	20	7.43	
	R	FPC-middle frontal gyrus	26	62	0	4.53	
663	L	Middle frontal gyrus	−36	48	16	7.44	0.000
510	R	IFG (p. triangularis)	36	32	28	7.19	0.000
	R	Middle frontal gyrus	34	36	28	7.02	
686	L	Insula lobe	−38	18	0	8.97	0.000
943	R	Insula lobe	36	18	4	7.63	0.000
285	L	Locus coeruleus	−4	−32	−14	5.38	0.000
	L	Locus coeruleus	−8	−26	−24	4.47	

*From left to right, the extent of the cluster in number of voxels (K; 2 × 2 × 2 mm^3^), the hemispheric lateralization (H; L, Left; R, Right), the estimated anatomical region [and Brodmann Area where available in the Anatomy Toolbox (Eickhoff et al., 2005)], the stereotactic coordinates (MNI), the local-maxima T-score and the cluster p-value, are reported for the regions that were more strongly activated by exploitative than explorative decision-making (top), and explorative than exploitative decision-making (bottom). Values shown in the Cluster p-value column survive a statistical threshold of p < 0.05 corrected for multiple comparisons. CA, Cornu Ammonis; IFG (p. Orbitalis), pars orbitalis of the Inferior Frontal Gyrus; IFG (p. Triangularis), pars triangularis of the Inferior Frontal Gyrus; SMA, Supplementary Motor Area; SUB, Subiculum; vmPFC, ventromedial prefrontal cortex*.

In order to examine the role of specific regions of interest (ROIs) in efficient exploration/exploitation tradeoffs, we extracted BOLD signal estimates from 8 mm radius spheres centered on functionally independent MNI coordinates reported in the previous studies listed below. All spheres were defined using the SPM-toolbox Marsbar (http://marsbar.sourceforge.net) and parameter estimates for both explorative and exploitative choices were extracted using the toolbox REX (http://web.mit.edu/swg). A dACC ROI was defined using the center of mass (−2, 21, 34) of the ACC coordinates reported by Kolling et al. (2012). Five ROIs for vmPFC (−3, 33, −6), bilateral FPC (left: −27, 48, 4; right: 27, 57, 6), and bilateral IPS (left: −29, −33, 45; right: 39, −36, 42) regions previously associated with exploitative vs. explorative choice were centered on the coordinates reported by Daw et al. (2006). An additional ROI in right FPC was created from the coordinates (36, 54, 0) of a region reported by Boorman et al. (2009) to track the value of alternative courses of action and predict effective switching between exploitation and exploration. Lastly, ROIs for the locus coeruleus were defined on the basis of anatomical criteria because the evaluation of Blood-Oxygen-Level-Dependent (BOLD) responses in the brainstem suffers from intrinsic limitations (Astafiev et al., 2010). Specifically, the centers of left (−4, −37, −23) and right (5, −37, −23) locus coeruleus ROIs were defined as the center of mass of representative coordinates from a neuroanatomical MR study (Keren et al., 2009) that localized the left and right locus coeruleus by assessing the distribution of neuromelanin, a pigment that is produced in noradrenergic neurons (Zucca et al., 2006).

We conducted two analyses on the data from the ROIs defined in the preceding paragraph. First, we conducted the same 2 × 2 ANOVA (choice type × group) used for the whole brain analysis within the FPC region shown by Boorman et al. (2009) to correlate brain activity with a measure of optimal switching (exploit vs. explore) behavior across subjects. Second, we conducted a multiple regression analysis to test whether activity in these independently defined ROIs was associated with individual differences in efficiency scores in addition to the group differences in efficiency identified at the behavioral level. We included activity in all independently defined ROIs that showed significant differences between explore and exploit trials (see Table 4) together with an indicator variable for entrepreneur group membership in a multiple linear regression.

**Table 4:** **Regions-of-Interest analyses in independent coordinates**.

**Source**	**ROI**	**Effect**	***t*-value**	**DF**	***p*-value**
Daw et al., 2006	vmPFC	Exploit vs. Explore	5.094	98	8.5E-07
	L FPC	Explore vs. Exploit	2.074	98	0.020
	R FPC	Explore vs. Exploit	2.635	98	0.005
	L IPS	Explore vs. Exploit	4.065	98	4.86E-05
	R IPS	Explore vs. Exploit	6.179	98	7.39E-09
Boorman et al., 2009	R FPC	Explore vs. Exploit	2.17	98	0.032
Kolling et al., 2012	dACC	Explore vs. Exploit	4.479	98	1.01E-05
Keren et al., 2009	LC	Explore vs. Exploit	1.631	98	0.053

*Regions-of-interest (ROIs) analyses in the coordinates previously associated with exploitative choice, i.e., ventromedial prefrontal cortex (vmPFC), and explorative choice, i.e., left and right frontopolar cortex (FPC) and intraparietal sulcus (IPS), dorsal sector of anterior cingulate cortex (dACC) and locus coeruleus (LC). L, left; R, right; DF, degrees of freedom*.

## Results

### Behavioral performance

**Figure 2:**
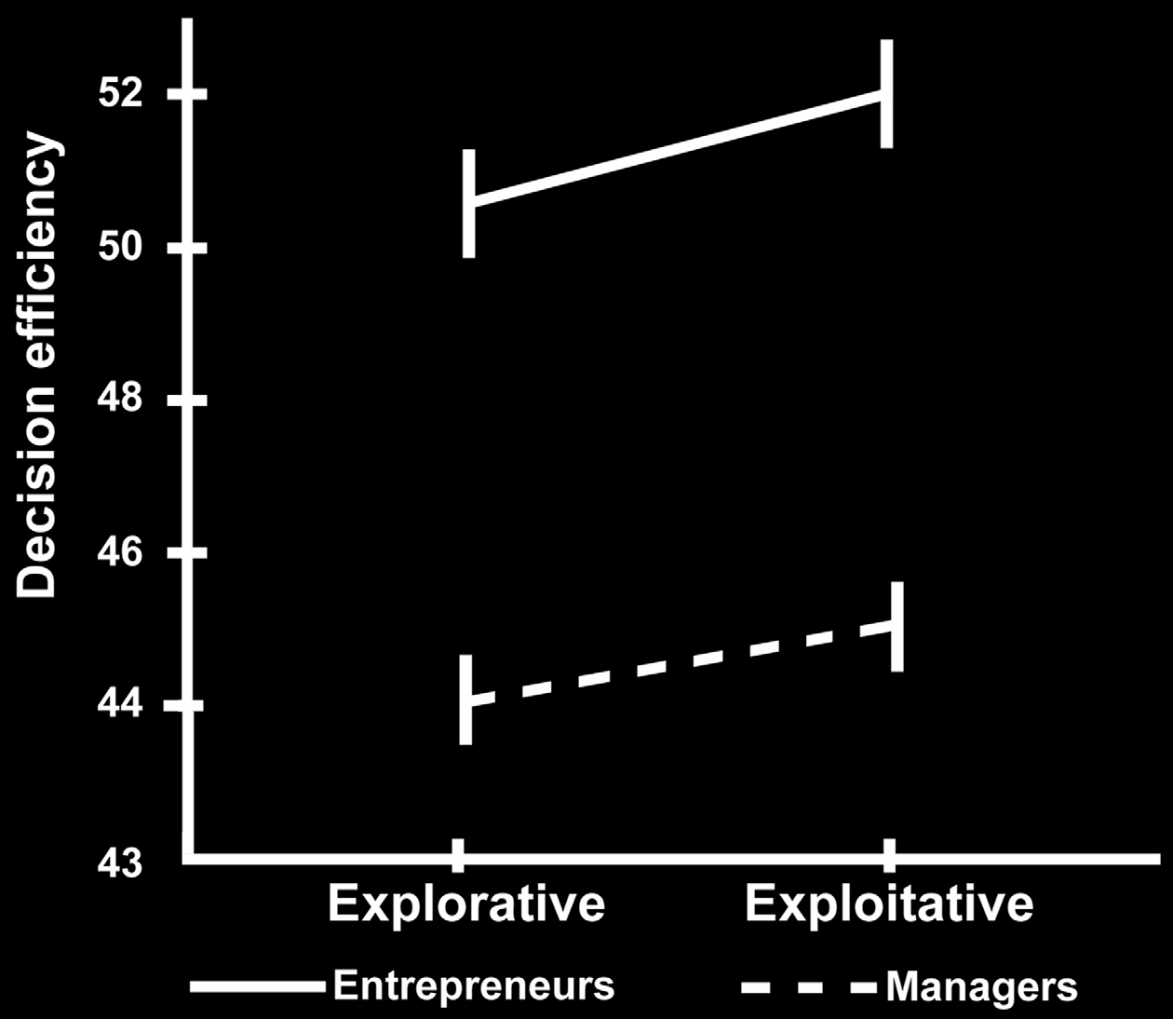
**Behavioral results**. Decision-making efficiency in explorative and exploitative choices for the group of managers (dotted line) and entrepreneurs (simple line). The vertical bars reflect the standard errors.

First, we found that the modeling of participants' behavior in the 4-armed bandit task with a Kalman filter and softmax choice rule fits equally well to the manager (negative log likelihood = 7.2e3) and entrepreneur groups (negative log likelihood = 7.2e3), with no significant group differences in their softmax exploration model parameters [Entrepreneurs mean = 0.19, *SD* = 0.8; Managers mean = 0.17, *SD* = 0.09; *t*_(48)_ = 1.04, *p* = 0.3]. Therefore, we used the model parameters estimated across all subjects as a single group to define exploration and exploitation choices. Next, we tested the hypothesis that, compared with the managerial control group, entrepreneurs would show higher choice efficiency, operationalized as performance in the bandit task divided by the time spent making the decision. To this purpose we used a 2 × 2 ANOVA over choice type (explore vs. exploit) and group (entrepreneurs vs. managers), with total payoff divided by response time as dependent variable. There were main effects of choice type on our measure of efficiency [*F*_(1, 48)_ = 9.977, *p* < 0.01], and group [*F*_(1, 48)_ = 4.155, *p* < 0.05], but no significant interaction (Figure 2). In particular, entrepreneurs displayed more efficient decision processes compared with managers, in that they obtained the same profit (points) in a significantly faster time (Entrepreneurs' efficiency = 47.180, *SD* = 5.840; Managers' efficiency = 41.625, *SD* = 10.615, *p* < 0.05).

For completeness, we also tested the individual components of our efficiency measure separately. Note that these analyses are conducted for the sole purpose of understanding how the individual components of our efficiency measure are related to choice type and group, rather than tests of independent hypotheses. We performed two additional 2 × 2 ANOVAs using either points earned or reaction times as dependent variable. There was a main effect of choice type on earnings [*F*_(1, 48)_ = 2306.636, *p* < 0.0001; Table 2]. However, there was no main effect of group [*F*_(1)_ = 0.494, *p* = 0.486] or any interaction between group and choice [*F*_(1)_ = 1.799, *p* = 0.186] on earnings. This earnings difference between choices is to be expected because exploratory choices are defined from the computational model as those choices where the subject selected a slot machine other than the one believed to currently have the highest payout. Lastly, the 2 × 2 ANOVA on reaction times revealed main effects of both choice type [*F*_(1, 48)_ = 18.439, *p* < 0.0001], and group [*F*_(1, 48)_ = 6.518, *p* < 0.05], but no significant interaction. These tests of the individual factors comprising our measure of efficiency show that the group difference in efficiency is driven by the Entrepreneur group's ability to obtain equal earnings in less time than the Manager group (see Table 2).

### Neuroimaging analyses of experts in an exploration-exploitation task

**Figure 3:**
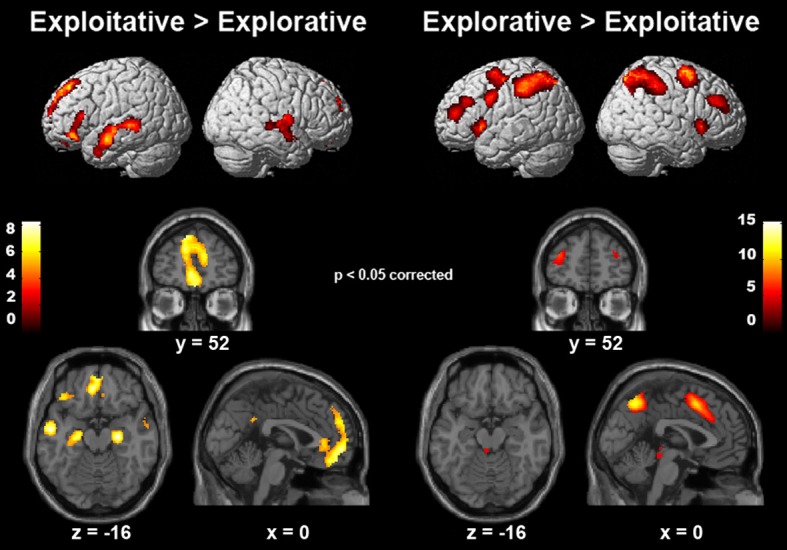
**Cerebral regions differentially activated by explorative and exploitative choices**. The cerebral regions that were more strongly activated by exploitative than explorative choices (left), and by explorative than exploitative choices (right).

We employed event-related *f*MRI analyses to investigate differences in neural activation while entrepreneurs and managers made exploitative vs. explorative choices. We first tested the effect of choice (explore vs. exploit), group (entrepreneurs vs. managers) and their interaction using a whole-brain 2 × 2 ANOVA, and found a significant main effect of choice. Compared with explorative choices, exploitative ones elicited significantly stronger activations of mesocortico-limbic regions, namely medial prefrontal cortex (extending dorsally from the vmPFC to the paracingulate cortex) and hippocampus (cornu ammonis and subiculum) bilaterally (Figure 3 left; Table 3-top). In contrast, explorative decision-making elicited significantly stronger activations of bilateral parietal and frontal regions, as well as of the locus coeruleus (Figure 3 right; Table 3-bottom). In the parietal cortex, activations extended from the temporo-parietal junction (TPJ) to the inferior parietal lobule, IPS, and superior parietal lobule. Frontal activations involved bilaterally the frontal eye fields (FEF), middle frontal gyrus, ventral fronto-insular cortex (VFC) and FPC. In the medial surface of the brain, the dorsal sector of anterior cingulate cortex (dACC; rostral cingulate zone) and pre-supplementary motor area (pre-SMA) were also activated. Neither the main effect of group, nor the interaction between choice type and group, survived correction for multiple comparisons at the whole brain level.

Next, we tested whether the better decision efficiency displayed by entrepreneurs compared to managers in our task that requires tracking the values of multiple alternative actions may reflect group differences in FPC activity. We focused on an FPC ROI that Boorman et al. (2009) previously associated with tracking evidence for alternative actions and individual differences in effective behavioral switching. In line with our hypothesis, activity in this region displayed a main effect of choice type (see Table 4) and a significant interaction between choice-type and group [*F*_(1, 48)_ = 4.0890, *p* < 0.05]. This interaction was driven by a greater increase in activity for explorative vs. exploitative choices in the more efficient entrepreneur group (mean = 0.67, *SD* = 1.60) compared to managers (mean = −0.15, *SD* = 1.18).

**Figure 4:**
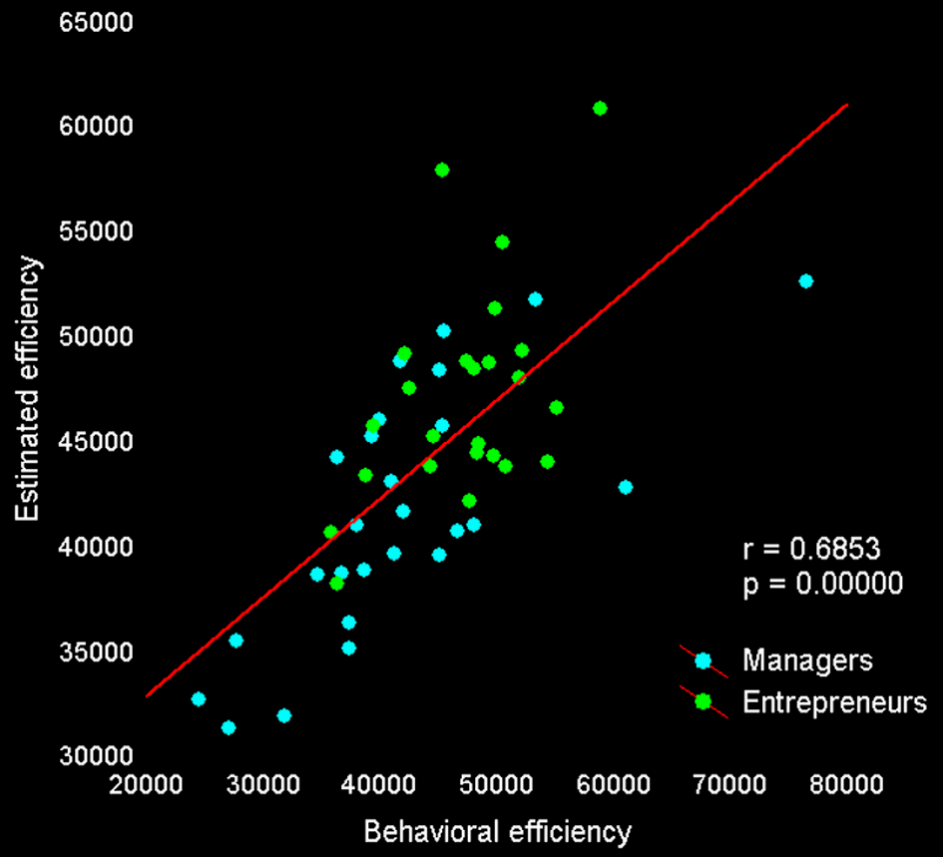
**Behavioral and predicted efficiency scores**. The relationship between *behavioral* decision-making efficiency (payoff divided by response time) during *f*MRI scanning and *estimated* efficiency, based on the activity of seven regions reported in previous studies and showing a significant difference between exploitative and explorative choice [vmPFC, bilateral FPC, and IPS from Daw et al. (2006); right FPC by Boorman et al. (2009); dorsal sector of anterior cingulate cortex by Kolling et al. (2012)].

Lastly, we tested whether activity in our set of *a priori* ROIs was associated with individual differences in decision efficiency scores. The overall multiple linear regression including regressors for group membership and activity during explore and exploit trials in seven ROIs (see Table 5) explained a significant amount of the variance in individual decision efficiency scores [*R*^2^ = 0.47, adjusted *R*^2^ = 0.24; *F*_(15, 34)_ = 2.01, *p* < 0.05; see Figure 4]. Contrasts for individual regressors revealed that activity in FPC was significantly related to decision efficiency across subjects and that there were marginally significant effects of vmPFC and dACC (Table 5).

**Table 5:** **Multiple regression coefficients and statistics**.

**Coefficients**	**Estimate**	**Std. Error**	***t*-value**	***P*r(>|*t*|)**
(Intercept)	30914.91	3825.79	8.081	2.02e-09^***^
ENT	8536.70	2855.16	2.990	0.00516^**^
Right bFPC-explore	1192.04	1054.74	1.130	0.26631
Left dFPC-explore	2146.77	2339.89	0.917	0.36536
Right dFPC-explore	−1933.72	1153.99	−1.676	0.10297
dACC-explore	846.44	1112.46	0.761	0.45198
vmPFC-explore	−1459.22	852.69	−1.711	0.09613^*^
Left IPS-explore	2399.80	2269.55	1.057	0.29779
Right IPS-explore	−65.39	1348.72	−0.048	0.96162
Right bFPC-exploit	1587.48	1163.16	1.365	0.18128
Left dFPC-exploit	−3448.30	3426.77	−1.006	0.32139
Right dFPC-exploit	5725.48	2078.71	2.754	0.00938^**^
dACC-exploit	2933.60	1601.67	1.832	0.07579^*^
vmPFC-exploit	−119.40	1237.02	−0.097	0.92367
Left IPS-exploit	−1022.54	3286.96	−0.311	0.75763
Right IPS-exploit	−2436.26	2216.64	−1.099	0.27946

Ent, entrepreneurs; bFPC, frontopolar cortex in Boorman et al. (2009); vmPFC, dFPC, and IPS, ventromedial prefrontal cortex, frontopolar cortex, and intraparietal sulcus in Daw et al. (2006); dACC, dorsal sector of anterior cingulate cortex in Kolling et al. (2012). Significance codes:

*** <0.0001;

** <0.01;

* *<0.1*.

## Discussion

We addressed the neural bases of individual differences in decision-making efficiency by comparing brain activity underlying exploitative vs. explorative decision-making in matched groups of entrepreneurs (who manage the organization they founded based on their venture idea) and managers (specialized in making strategic decisions but with no venture experience) (Shane and Venkataraman, 2000; Shane, 2006; Ács and Audretsch, 2010).

In line with previous reports (Daw et al., 2006; Boorman et al., 2009), both whole-brain and ROIs results confirmed that exploitation and exploration are associated with the activation of different brain regions. Exploitative choices recruit ventromedial prefrontal activations involved in reward anticipation (Tobler et al., 2007) and tracking the value of the current choice (Boorman et al., 2009; Kolling et al., 2012). On the contrary, explorative choices engage fronto-parietal regions, alongside the dorsal sector of anterior cingulate cortex (dACC) and locus coeruleus, associated with executive and attentional control (Corbetta and Schulman, 2002; Boorman et al., 2009). We note that the difference between explore and exploit choices in LC was only marginally significant in our dataset, perhaps because of high levels of noise in the BOLD signal near this regions (Astafiev et al., 2010). This pattern of activation suggests an organization of the various brain regions that have been proposed to optimize aspects of decision performance into a coherent functional framework. A pivotal role in choice optimization has been attributed to noradrenergic activity in the locus coeruleus (Usher et al., 1999; Doya, 2008), although with variable interpretations. One account supports the role played by either the tonic or phasic functioning modes of the LC-NE system, favoring exploitation or exploration, respectively (Aston-Jones and Cohen, 2005). An alternative model suggests that noradrenergic activity signals so-called unexpected uncertainty (Yu and Dayan, 2005), i.e., sudden environmental switches indicating that learning must be re-started because outcome contingencies have changed. Regardless of a specific interpretation of the underlying computations, the noradrenergic involvement in driving exploration requires that information about rewards and costs associated with the current choice be fed into the LC. This is likely to depend on frontal projections from vmPFC and dACC, the most prominent among descending cortical projections to LC (Rajkowski et al., 2004; Zhu et al., 2004), with the former suggested to encode the relative value of the current decision, and the latter to signal the value of exploring the environment (Hayden et al., 2011; Kolling et al., 2012). Along with the FPC, associated with tracking the value of alternative choices (Daw et al., 2006; Boorman et al., 2009) activity in dACC may thus play a key role in exploration. This region, indeed, likely drives transitions from the phasic to the tonic LC mode, resulting in disengagement from the current choice, distributed attention and, finally, search for new options (i.e., explore) (Aston-Jones and Cohen, 2005). Overall, our current results together with previous findings support the role of this network of regions (FPC, vmPFC, dACC, IPS, and the LC-NE system) in the optimization of decision performance.

Here we assessed whether activity in these regions subserving exploration (vs. exploitation) is specifically associated with the higher decision-making efficiency displayed by entrepreneurs compared with managers. As expected, the two groups were comparable in terms of total payoff, indicating that the observed differential brain activations were not driven by major differences in performance with regard to maximizing payoffs (Price and Friston, 1999; Murphy and Garavan, 2004). Entrepreneurs, however, were able to get the same result in less time, indicating more efficient choice behavior in the uncertain context of the bandit task. Moreover, we found a group-specific neural signature of entrepreneurs' higher decision-making efficiency in the FPC, a key region for explorative choice. We also found that activity in FPC, as well as vmPFC and dACC to a lesser extent, explained individual differences in decision efficiency after controlling for entrepreneur vs. manager status.

Overall, these data support the association between individual differences in efficient decision-making in a classical task of exploration/exploitation choice and activity in a network subserving explorative behavior. Our results thus suggest that expert decision-making success may be enhanced by the individuals' ability to track the evidence in favor of constantly evolving alternative options, and in disengaging attention from current reassuring options, both mechanisms leading to more efficient decision-making. These same skills are likely to promote success in entrepreneurial endeavors that require attending to rapidly changing, and unforgiving, environmental circumstances.

While highlighting a coherent picture of the neural bases of individual differences in efficient decision-making performance, the present findings about activity in the regions underpinning explorative behavior raise a crucial question that awaits supporting empirical evidence. Are such differences in neural activation and choice efficiency the consequence of a self-selection process leading to a career path in line with a natural predisposition, or rather of brain plasticity phenomena resulting from daily work challenges? Supporting the former view, some variance in the tendency of becoming an entrepreneur can be attributed to genetic inheritance (Nicolau et al., 2008), and differences in explorative behavior have been associated with polymorphisms in the COMT gene (Frank et al., 2009). Moreover, the COMT Met/Met genotype has been associated with novelty seeking behavior (Golimbet et al., 2007) and enhanced executive functioning, including attentional control (Egan et al., 2001). Yet, the possible role of neuroplasticity processes linked to entrepreneurial traits that can be learned or improved with experience remains an open question and an important topic for future investigation. The extent to which entrepreneurial abilities to switch efficiently from exploitation to exploration and back can be a priori assessed, as well as developed via specific training and practice, has significant implications for the enhancement of innovation outcomes and overall competitiveness at the organizational, national, and international levels. Be it the result of a genetic predisposition, or of environmental pressures associated with experience, individual differences in computational processes performed by the FPC seem to underpin the ability of rapidly take decisions under uncertainty that supports entrepreneurial success.

## Conclusion

Our findings contribute to the understanding of the neural mechanisms supporting the ability to attend to environmental opportunities, and track evidence to decide when to disengage from exploitation and explore novel alternatives. These results suggest that entrepreneurship is associated with the individuals' ability in tracking the evidence in favor of switching between alternatives, and in disengaging attention from current reassuring options, both mechanisms leading to more efficient decisional switching patterns.

### Conflict of interest statement

The authors declare that the research was conducted in the absence of any commercial or financial relationships that could be construed as a potential conflict of interest.
